# Bridging Gaps in Holistic Rehabilitation After Critical Illness: A Systematic Review

**DOI:** 10.3390/healthcare13182324

**Published:** 2025-09-16

**Authors:** Anna Korompeli, Kalliopi Kydonaki, Pavlos Myrianthefs

**Affiliations:** Department of Health Science, Faculty of Nursing, National and Kapodistrian University of Athens, 11527 Athens, Greece; ckydonaki@nurs.uoa.gr (K.K.); pmiriant@nurs.uoa.gr (P.M.)

**Keywords:** holistic care, intensive care, patient outcome, wellness

## Abstract

Background: Holistic care in the Intensive Care Unit (ICU) addresses the full spectrum of patient needs—physical, emotional, psychological, social, spiritual, and environmental—to support recovery and improve long-term outcomes after critical illness. Objective: This systematic review aimed to synthesize evidence on the effectiveness of holistic care interventions across these six dimensions of wellness in adult ICU patients. Methods: A systematic search of PubMed, Scopus, and Web of Science was conducted following PRISMA guidelines. The SPICE framework was used to define the scope (Setting: ICU; Perspective: patients; Intervention: holistic care; Comparison: standard care; Evaluation: multi-dimensional outcomes). Studies published in English between 1999 and 2024 were included. Methodological quality was appraised using Joanna Briggs Institute (JBI) tools. Results: Seven studies, comprising randomized controlled trials, observational, and mixed-methods designs, met the inclusion criteria. The interventions were diverse, encompassing corporeal rehabilitation, spiritual care toolkits, reflexology, early physical therapy, patient diaries, and family involvement. A narrative synthesis of these heterogeneous studies suggested potential benefits and high acceptability for various patient-centered outcomes. Conclusions: The limited but promising evidence indicates that holistic care interventions may contribute positively to ICU patient recovery. The findings underscore the need for more robust, high-quality research to conclusively determine their efficacy and support their integration into standard critical care practice.

## 1. Introduction

Holistic care in the intensive care unit (ICU) is a comprehensive philosophy that transcends the traditional biomedical model. It is defined as an approach that emphasizes the need to address the full spectrum of patients’ and families’ needs—encompassing the physical, emotional, psychological, social, spiritual, and environmental dimensions of well-being to support recovery and improve long-term outcomes after critical illness [1]. This approach acknowledges that critically ill patients face not only physiological challenges but also profound psychological distress, social isolation, and existential concerns [2].

Several reviews have indicated that the implementation of holistic care practices can lead to improved patient outcomes, including reduced anxiety, enhanced coping mechanisms, and increased overall satisfaction with care [3,4,5,6]. This approach is operationalized through practices such as early mobilization and physical rehabilitation to address physical deconditioning [7], integrating psychological support and delirium management to protect mental health [8], actively involving families in care decisions and communication [9], and addressing the spiritual and existential needs of patients [10,11].

To operationalize and systematically evaluate this multifaceted concept, this review adopts the Six Dimensions of Wellness model as its theoretical framework [1,12]. This model was selected because it provides a structured, comprehensive, and patient-centered lens through which to view recovery, moving beyond a narrow focus on physical rehabilitation to encompass the complete human experience of illness and healing. Its origins in health promotion make it particularly suitable for framing interventions aimed at improving long-term outcomes and quality of life after critical illness. For the purposes of this review, these dimensions are defined as follows, with particular attention to the interrelated yet distinct nature of emotional, psychological, and cognitive wellness:

- Physical wellness emphasizes the physiological aspects of health, including mobility, strength, and overall physical function. Early mobilization through passive and active exercises, respiratory therapy, and nutritional support is crucial to prevent complications such as muscle atrophy and pressure ulcers [13].
- Emotional wellness refers to the affective domain, encompassing the ability to recognize, express, and manage a full range of emotions, from anxiety and fear to hope and resilience. For ICU patients, this dimension is vital for processing the traumatic experience of critical illness. Providing emotional support through therapeutic communication, counseling, and family involvement helps patients navigate this distress [14].
- Psychological wellness is conceptualized here as a broader construct that includes both mental health and cognitive functioning. This integrated view is justified by the profound interplay in the ICU between a patient’s mental state (e.g., the presence of anxiety, depression, or delirium) and their cognitive capacities (e.g., attention, memory, executive function). Critical illness and the ICU environment can simultaneously impair both, making their combined consideration essential for a holistic recovery model. Interventions such as mental health support, delirium assessments, and orientation activities are therefore grouped under this dimension to promote overall psychological and cognitive recovery [15].
- Social wellness highlights the importance of relationships and social support networks in recovery. Interventions such as family involvement in care, communication facilitation, and participation in support groups (e.g., ICU Steps in the UK) strengthen social support systems [16].
- Spiritual wellness involves finding meaning and purpose in life, particularly significant for patients facing life-threatening conditions. Integrating spiritual care includes addressing patients’ spiritual needs, discussing values and beliefs, and offering access to chaplaincy services, meditation, or personal reflection [10,11].
- Environmental wellness considers how surroundings affect health and well-being, including the physical and emotional atmosphere of the ICU. Creating a healing environment by minimizing noise, ensuring privacy, optimizing lighting, and providing comfort measures enhances recovery and fosters a sense of safety [17].

Despite a growing body of literature supporting the benefits of holistic care in the ICU [18,19], a significant gap remains in understanding how the integration of these six dimensions of wellness can be systematically implemented and evaluated in diverse ICU settings to optimize patient outcomes. This review aims to bridge that gap by synthesizing evidence structured around this comprehensive framework.

## 2. Materials and Methods

### 2.1. Research Questions

This systematic review aimed to evaluate the impact of implementing holistic care—encompassing physical, emotional, psychological, social, spiritual, and environmental dimensions—compared to standard nursing care on patient outcomes. These outcomes included measures of physical recovery, emotional well-being, and overall quality of life during and after ICU admission.

### 2.2. Study Design

A comprehensive methodological framework for systematic reviews, as described by Higgins et al., 2019 [20], was employed to permit the inclusion of all research designs, both experimental and non-experimental. The review process was designed and conducted in accordance with the PRISMA statement.

The protocol for this systematic review was registered with the International Prospective Register of Systematic Reviews (PROSPERO) under registration number CRD420251090811.

### 2.3. Definition of Variables

The Setting–Perspective–Intervention–Comparison–Evaluation (SPICE) framework was used to define variables and inclusion and exclusion criteria (Table 1).

### 2.4. Search Methods

A systematic search was conducted in November 2024 across three major databases: PubMed (including MEDLINE), Scopus, and Web of Science. The search was limited to studies published in English between 1999 and 2024. The year 1999 was selected as the start date to capture literature published after the influential Institute of Medicine (IOM) report “To Err is Human” (2000), which catalyzed a global shift towards patient safety and quality of care, including a greater focus on patient-centered approaches in all medical specialties, such as the holistic model of care in the ICU [21]. This period also encompasses the modern evolution of critical care rehabilitation and the formal recognition of Post-Intensive Care Syndrome (PICS), ensuring the review captures the most relevant and contemporary evidence [22]. Hand searches were also performed to capture additional relevant studies. The following search terms were used, employing Boolean operators and truncations: **“holistic care,” “intensive care,” “patient outcome,”** along with specific terms related to each wellness dimension (spiritual, mental, physical, emotional, psychological, social, environmental) (Table 2).

Articles were screened according to the inclusion and exclusion criteria. Authors of studies with only abstracts available were contacted for full-text versions. Grey literature, editorial comments, and conference abstracts were excluded.

### 2.5. Methodological Quality Appraisal

Methodological quality was appraised using the Joanna Briggs Institute (JBI) Critical Appraisal tools guidelines [23]. The JBI tool enabled a rigorous assessment of methodological strengths and weaknesses across study types. No studies were excluded based on quality ratings.

### 2.6. Data Abstraction

Two researchers (AK, KK) independently extracted data using a structured data extraction tool based on the Cochrane guidelines. Extracted variables included author, year, country, design, sample size, demographics, ICU characteristics, cohort details, intervention description, comparison conditions, outcome measures, primary and secondary results, study limitations, and JBI quality scores. Disagreements were resolved through consultation with a third reviewer (PM).

### 2.7. Data Analysis

Due to the significant clinical and methodological heterogeneity observed across the included studies—including variations in intervention types, study populations, settings, and outcome measures—a meta-analytic approach was deemed inappropriate. Consequently, an inductive thematic analysis and narrative synthesis, following the Cochrane Consumers and Communication Review Group’s guidance, were conducted. This approach allowed for the exploration of relationships and themes within and between studies, providing a comprehensive qualitative understanding of the effectiveness and implementation of holistic care interventions.

### 2.8. Ethical Considerations

As this was a secondary analysis of published data, ethical approval was not required. All included studies had previously obtained ethical approval.

## 3. Results

**Figure 1:**
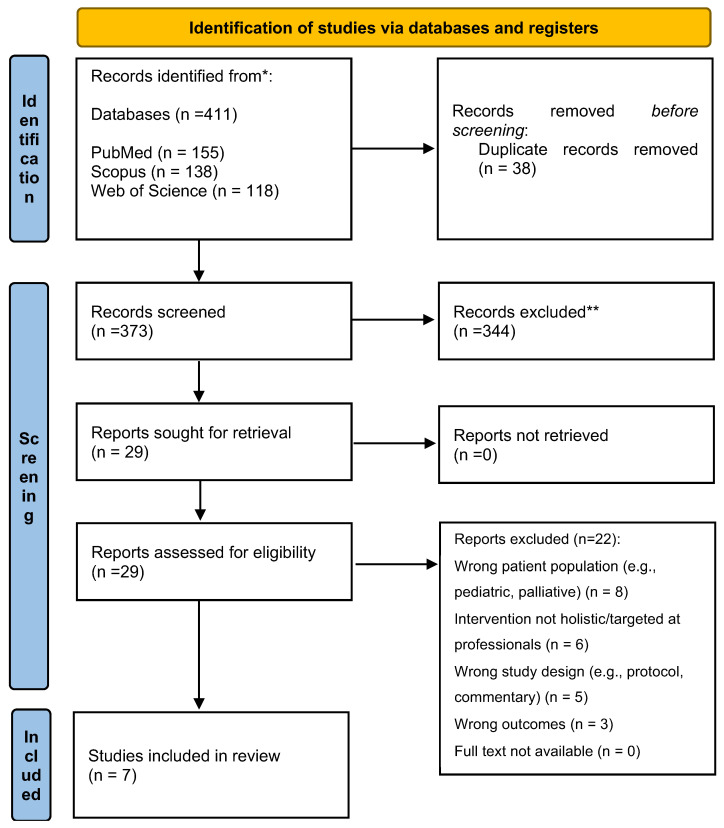
PRISMA 2020 flow diagram of the study selection process. Note: The systematic search was conducted in November 2024. Records were excluded during screening primarily for not involving an ICU setting, not testing a holistic care intervention, or not measuring relevant patient outcomes.

The study selection process is detailed in the PRISMA 2020 flow diagram (Figure 1). A systematic search across three databases (PubMed, Scopus, Web of Science) and supplemental hand-searching yielded 411 total records. After removing 38 duplicates, 373 unique records were screened based on their titles and abstracts. Of these, 344 records were excluded, leaving 29 studies for full-text review. Upon full-text assessment, 22 studies were excluded with reasons, resulting in 7 studies that met all eligibility criteria and were included in the qualitative synthesis of this systematic review.

Some studies incorporated elements tangential to these domains (e.g., family involvement as a component of a psychological intervention [24] but none had the explicit objective of evaluating their isolated impact on social connectedness or the healing environment. Consequently, the following synthesis of results is structured around the four dimensions for which direct interventional evidence was found. The absence of studies targeting social and environmental wellness is in itself a crucial result of this review, highlighting a substantial gap in the current literature that warrants prioritization in future research.

The studies were generated from the USA, France, UK, and Turkey. A variety of research designs were used, and most studies were conducted in a single ICU setting, and one study [25] was conducted in post-ICU weaning and rehabilitation center (Table 3). 

The methodological quality of the included studies was appraised using the relevant Joanna Briggs Institute (JBI) critical appraisal tools. The results of this appraisal are summarized in Table 4, indicating a variable level of methodological rigor across the studies. All studies were retained for analysis to provide a comprehensive overview of the existing literature on holistic care interventions.

This review encompassed a variety of studies aiming to enhance the understanding and application of holistic care interventions for critically ill patients. The findings from these diverse studies are presented below to provide an exploratory synthesis of the existing literature; however, the heterogeneity in design, intervention, and outcomes precludes definitive conclusions on efficacy.

Our systematic review was designed and conducted with the explicit aim of synthesizing evidence across all six dimensions of wellness (physical, emotional, psychological, social, spiritual, and environmental) as defined by our theoretical framework. The search strategy was intentionally broad and inclusive of terms related to all these dimensions (e.g., “social,” “environmental”) to ensure comprehensive coverage of the literature.

However, upon full-text screening and data extraction, we identified a significant evidence gap: no studies were found that primarily investigated interventions targeting social wellness (e.g., building new support networks beyond family) or environmental wellness (e.g., structural modifications to the ICU healing environment) as standalone, evaluable concepts. While some included interventions, such as family involvement [24], had social components, their primary objective and outcome measures were not focused on evaluating the isolated impact on social connectedness or the healing environment.

Therefore, the synthesis of results was structured around the four dimensions (physical, psychological, emotional, and spiritual) for which direct interventional evidence was found.

All included studies involved critically ill patients admitted to the intensive care unit (ICU) [24,25,26,27,28,29,30]. Kincheloe et al. (2018) [27] reported a mixed sample of nurses, patients, and family members, whereas Black et al. (2011) [24] included both patients and families. The sample sizes of patients and family members ranged from 50 to 297 participants across the studies. All studies included patients who had spent more than 24 h in an ICU.

The methodological quality of the included studies, as appraised by JBI tools (Table 4), was variable. This heterogeneity in quality must be considered when interpreting the findings. For instance, the randomized controlled trials by Bulut et al. [30] and Korhan et al. [29], which had the highest JBI scores (9/13 and 8/13, respectively), provide more robust evidence for the efficacy of spiritual care interventions and reflexology. In contrast, the promising findings from observational studies with lower scores and inherent design limitations, such as the lack of a control group in Bourgeon-Ghittori et al. [26] (6/8) and Lemyze et al. [25] (6/10), must be interpreted with greater caution, as they are more susceptible to bias and confounding. The following synthesis presents the results from all studies but notes where the strength of the evidence is tempered by methodological concerns.

### 3.1. Interventions for Physical Wellness

The studies in this section apply a holistic approach by demonstrating that interventions focused on physical rehabilitation also yield significant benefits in emotional and psychological domains, moving beyond a narrow focus on functional recovery. This category explores integrated rehabilitation methods designed to promote physical and functional recovery in critically ill patients. Two recent studies illustrate the effectiveness of patient-centered approaches that transcend conventional clinical routines. Bourgeon-Ghittori et al. (2022) [26] conducted a study involving 297 ICU patients to evaluate the impact of Corporeal Rehabilitation Care (CRC)—a multisensory, esthetic care intervention combining sensory stimulation and empathetic communication. Immediately after CRC sessions, patients exhibited significant reductions in stress. Physical benefits, including reduced dyspnea and respiratory rate, were also observed. Among the 287 evaluable patients, 66% were classified as responders. Notably, CRC responders had significantly lower 28-day mortality (4%) compared to non-responders. Median satisfaction and usefulness scores reached 10/10.

Lemyze et al. (2022) [25] assessed outcomes in 50 patients recovering from severe COVID-19 pneumonia and chronic critical illness using a dual strategy—early intensive physical rehabilitation combined with a structured decannulation protocol. Results showed significant improvement in functional independence, with ADL scores increasing from 0 to 6. The 6 min walk test distance rose, frailty scores decreased, and HADS scores improved. The median rehabilitation period was 25 days, following an average ICU stay of 39 days.

### 3.2. Interventions for Psychological and Emotional Wellness

These studies exemplify a holistic approach by implementing strategies that address the profound psychological impact of critical illness, recognizing that mental and emotional recovery is as crucial as physical healing. Focused on enhancing the psychological well-being and recovery of critically ill patients, this category includes studies that explore interventions and their impact on mental health. This study by Pattison et al., 2019 [28] investigates the use of patient diaries in critical care settings with the aim to explore how patient diaries contribute to psychological recovery. Diaries were maintained by healthcare professionals and families during ICU admission and returned to patients during follow-up, offering a narrative bridge to reconnect patients with a fragmented or lost ICU experience. A total of 95% of participants found the diaries helpful, and 90% reported they helped fill in memory gaps. Patients used the diaries to reconstruct timelines and interpret their experiences, which supported emotional adjustment and reduced disorientation post-discharge. Despite modest changes in quantitative outcomes—mean PTSS-14 scores rose from 26.7 to 31.3 and EQ-5D VAS scores declined from 79.1 to 71.5 over 12 months—these changes were not statistically significant, suggesting complexity in interpreting these metrics in this population. Qualitative data from the study highlighted the value of the diaries in reconstructing the patient’s narrative and aiding emotional processing, with contributions from both healthcare staff and family members being a key component.

Another dimension of critically ill patients’ mental health is the effects of family participation in the psychological care. The primary aim of the study of Black et al., 2011 [24] is to determine how involving family members can influence the level of patient delirium and promote psychological recovery. Incorporating family engagement into psychological care represents a holistic strategy to buffer critically ill patients against psychological stressors. Black et al. (2011) [24] investigated the impact of nurse-facilitated family involvement. The intervention did not significantly reduce the incidence of delirium. Psychosocial SIP scores were significantly lower (indicating better well-being) in the intervention group across all time points. Korhan et al. (2014) [29] evaluated the effects of reflexology. Systolic BP, Diastolic BP, Heart Rate, and Respiratory Rate all decreased significantly across all 5 days in the reflexology group compared to controls. Significant improvements were also observed in sedation-related subscales: agitation, anxiety, sleep, and patient–ventilator synchrony. Sedation needs decreased in the intervention group. No significant change in consciousness levels between groups was reported.

### 3.3. Interventions for Spiritual Wellness

The interventions here operationalize holistic care by acknowledging and actively addressing the spiritual dimension of the human experience in critical illness, which is integral to complete well-being. Recognizing the importance of addressing the spiritual needs of patients and their families, this category includes studies focused on spiritual care initiatives in healthcare settings. The study by Kincheloe et al., 2018 [27] aimed to improve the delivery of spiritual care in acute healthcare environments through several specific objectives. Kincheloe et al. (2018) [27] sought to improve the provision of spiritual care (SC) in acute healthcare settings by introducing an evidence-based intervention: a Spiritual Care Toolkit. The study had several objectives. First, it aimed to develop a culturally inclusive SC toolkit containing items such as books, music, rosaries, and journals that could be used by patients, families, and nursing staff. Second, it assessed the baseline spiritual perspectives of nurses, patients, and families to identify differences that might influence SC delivery. Third, it evaluated the toolkit’s effectiveness in addressing spiritual needs and overcoming obstacles that hinder nurses from providing SC. Significant disparities were found between the spiritual perspectives of nurses and those of patients and families, with the latter groups displaying higher engagement in spiritual practices. Patients and families perceived the SC toolkit as effective, with 62% to 75%, rating the various resources as helpful. Nurses also reported increased confidence in providing SC, with statistically significant improvements in comfort, resource availability, and training following toolkit implementation. However, time constraints and communication discomfort remained unchanged.

The study of Bulut et al., 2023 [30] aimed to examine the effects of spiritual care interventions on the spiritual well-being, loneliness, hope, and life satisfaction of patients treated in an intensive care unit (ICU). The study investigated the impact of spiritual care interventions on ICU patients’ spiritual well-being, loneliness, hope, and life satisfaction. Conducted as a randomized controlled trial with 64 participants (32 in each group), the study employed the T.R.U.S.T. spiritual care model across eight sessions over four weeks. The intervention group exhibited a significant increase in Spiritual Well-Being compared to a non-significant change in the control group. Hope levels in the intervention group rose while the control group remained largely unchanged. Emotional loneliness decreased and social loneliness significant dropped. **Life Satisfaction** increased in the intervention group, with no significant change in the control group.

## 4. Discussion

This review was explicitly framed around the concept of holistic care, defined by its focus on the six dimensions of wellness. It is important to position this concept within the broader evolving paradigm of critical care, which increasingly emphasizes person-centered care (PCC) and humanizing care [19,31]. While these terms are frequently used interchangeably and are deeply synergistic, they possess nuanced distinctions. Holistic care provides the comprehensive framework of what needs to be addressed (the multi-dimensional needs of the patient). In contrast, PCC describes how to achieve this, by ensuring care is co-created, respectful of individual preferences, and guided by patient values [31]. The humanizing care movement explicitly aims to counteract the inherent dehumanization of critical illness and ICU technology by restoring patient personhood, narrative, and dignity [32]—a goal that is a direct consequence of applying holistic and person-centered principles. The interventions synthesized in this review, from patient diaries [28,33,34] to corporeal rehabilitation [26], are practical manifestations of this integrated philosophy: they use person-centered methods (e.g., co-creating a diary) to deliver holistic support (e.g., psychological and emotional wellness) with the ultimate aim of humanizing the ICU experience.

The primary objective of implementing holistic care is to transcend the goal of mere survival and positively influence the patient’s overall quality of life (QoL), both during the ICU admission and in the long term. Our research question explicitly aimed to evaluate this impact. The Six Dimensions of Wellness model provides a robust framework for this assessment, as it conceptualizes QoL not as a unitary outcome but as a multi-faceted construct comprised of physical, emotional, psychological, social, spiritual, and environmental well-being [8]. The findings of this review therefore must be interpreted as contributing to these different facets of QoL.

In the immediate context of the ICU admission, the interventions reviewed primarily impacted what can be termed health-related quality of life (HRQoL) by mitigating distress and improving comfort. For instance, the significant, immediate reductions in patient-reported stress, pain, and improvements in well-being following Corporeal Rehabilitation Care (CRC) [26] directly enhance the in-ICU experience, making it less traumatic and more humane. Similarly, reflexology’s reduction in physiological anxiety and sedation needs [29] improves comfort and safety during mechanical ventilation, a period often marked by fear and agitation. These interventions address the emotional and physical dimensions of wellness, directly contributing to a better immediate QoL by alleviating suffering and fostering a sense of safety.

The impact on long-term QoL after ICU admission is evidenced by interventions focused on functional and psychological recovery. The dramatic improvements in functional independence (ADL scores) and mobility (6MWT) demonstrated by Lemyze et al. [25] are critical determinants of long-term QoL, enabling patients to regain autonomy and reintegrate into their home lives. Furthermore, interventions like patient diaries [28] and structured family involvement [24] target the psychological and social dimensions, which are intrinsically linked to long-term well-being. By helping patients reconstruct their narrative and process trauma, diaries can mitigate the development of post-intensive care syndrome (PICS), a major detriment to long-term QoL. Improved psychosocial recovery scores suggest better capacity to resume social roles and activities, a core component of overall life satisfaction post-ICU.

The criteria used for screening were patients hospitalized in an Intensive Care Unit and those who received an intervention within the context of holistic care. The studies reviewed describe a range of interventions that have been explored for enhancing various dimensions of wellness. This focus is strongly supported by a recent large-scale cohort study by Cao (2025), which provides robust evidence that Medical-Integrated holistic nursing significantly improves patient outcomes, reduces complications, and enhances comprehensive healthcare for ICU patients [35]. The included studies were conducted in the USA, France, UK, and Turkey, focusing on holistic care interventions for critically ill patients in Intensive Care Units (ICUs). The studies employ various research designs and assess multiple dimensions of wellness, including physical, psychological, emotional, and spiritual well-being. A range of outcomes is measured in the studies, including physical/functional metrics [26], physiological [25,29], psychological [24,28,29], and spiritual [27,30]. 

The synthesis of evidence from this review suggests that holistic care interventions may positively impact various dimensions of patient recovery in the ICU. However, the strength of this evidence is moderated by the methodological quality of the included studies. The most methodologically robust findings, based on higher-quality RCT designs [25,29], support the effectiveness of structured spiritual care and reflexology in improving specific patient outcomes. The significant improvements in physiological stress parameters (e.g., pain, respiratory rate) and functional outcomes (e.g., ADL scores, 6 min walk test) following interventions like CRC [26] and early intensive rehabilitation indicate that non-pharmacological, multi-sensory approaches can effectively address the complex physical sequelae of critical illness. However, the observational nature of these studies [25,26] limits the ability to definitively attribute these improvements solely to the interventions, as they may be influenced by other aspects of care or natural recovery. The high patient satisfaction scores associated with these interventions further suggest their high acceptability.

The positive findings related to psychological and emotional wellness, though derived from heterogeneous interventions, point to a common theme: the importance of addressing the patient’s narrative and relational context. The high acceptability and perceived utility of patient diaries [28], despite non-significant changes in standardized quantitative metrics, may indicate that these tools aid in meaning-making and processing of traumatic ICU experiences—an outcome not fully captured by traditional scales. It is notable that this mixed-methods study [28] scored well on the JBI appraisal (7/8 for both qualitative and quantitative components), lending credibility to its findings on patient experience. Similarly, the significant improvement in psychosocial recovery scores with family involvement [30] underscores the critical role of the patient’s social ecosystem as a therapeutic resource, even when targeting outcomes like delirium remains challenging. The quasi-experimental design of this study [24], while scoring reasonably (7/9), lacked randomization, which is a limitation affecting the strength of causal inference.

The results related to spiritual wellness [27,30] demonstrate that structured interventions can effectively address this often-neglected dimension. The significant improvements shown in the high-quality RCT by Bulut et al. [30] provide strong evidence that spiritual distress is a modifiable factor in the ICU. This is further corroborated by the recent systematic review by Wirakhmi & Purnawan (2025), which concluded that structured spiritual care models are effective in improving both psychological and physiological outcomes for ICU patients [36]. The finding that nurses’ and patients’ spiritual perspectives often differ highlights a potential gap in care that toolkit-based interventions can help bridge. The lower JBI score for the quasi-experimental toolkit study [27] (6/9), primarily due to the lack of a control group, suggests its findings on feasibility and acceptability are promising but require confirmation through more controlled research.

A crucial finding of this review is the identified evidence gap. Despite our comprehensive search, we found no studies that primarily investigated interventions targeting social wellness (e.g., building new support networks) or environmental wellness (e.g., structural changes to the ICU environment) as defined by our framework. While some interventions, like family involvement [24], had social components, their primary aim was not to improve social connectedness itself. This represents a significant opportunity for future research.

It is critical to interpret these findings as a preliminary, exploratory map of the field. The synthesis indicates areas of promise that warrant further investigation rather than establishing evidence of consistent, generalizable effects

### Contribution to Education

The results of the studies underline a critical need to enhance healthcare education by incorporating psychological and spiritual care aspects of care into training programs for future healthcare professionals [37]. For instance, the findings from Pattison et al., 2019 [27] regarding patient diaries show that these tools can be pivotal in aiding psychological recovery. Therefore, educational programs should teach future clinicians how to implement and make the most of these diaries to help patients process their experiences.

Similarly, Black et al., 2011 [24] insights into the importance of family engagement in patient care highlight that healthcare education must address how to involve families effectively. Training programs can be better equipped to produce professionals who foster an environment that supports family involvement in healthcare in order to improve patient recovery.

Moreover, the development of modules around spiritual care toolkits demonstrated by Kincheloe et al., 2018 [27] showcases the importance of educating healthcare providers on the spiritual dimensions of care. Such training prepares professionals to approach patient care holistically, addressing not only physical ailments but also the emotional and spiritual needs of patients [38].

## 5. Recommendations for Clinical Practice and Future Research

The synthesis of evidence from this review allows for the formulation of specific, actionable recommendations for key stakeholders involved in the care of critically ill patients.

### 5.1. Recommendations for Nursing Practice

- Implement Structured Non-Pharmacological Interventions: Integrate evidence-based practices such as Corporeal Rehabilitation Care (CRC) sessions [26] or reflexology [29] into daily nursing care plans for anxious or agitated patients to reduce physiological stress and potentially decrease sedation requirements.
- Facilitate Family Integration: Adopt a structured approach to family involvement. Provide families with a simple guidebook on communicating with sedated patients and actively facilitate their participation in psychological care, as modeled by Black et al. [24].
- Provide Spiritual Care Resources: Utilize a readily available Spiritual Care Toolkit [27] containing multi-faith resources (e.g., sacred texts, meditation audio, prayer journals) to address spiritual distress. Nurses should receive basic training on how to introduce and use these resources sensitively.
- Initiate ICU Diaries: Lead the implementation of patient diaries within the ICU. Coordinate contributions from the healthcare team and family members to create a narrative that helps patients process their experience and fill memory gaps post-ICU [28].

### 5.2. Recommendations for Relatives and Family Members

- Engage in Guided Communication: Use provided guidance to talk to the patient about familiar topics, read to them, or play their favorite music, even if they appear non-responsive. This can provide comfort and psychological support [24].
- Participate in Diary Creation: Contribute to the patient’s ICU diary by writing simple entries about daily events, family news, or words of encouragement. This provides a crucial personal perspective for the patient to reflect on later [28].
- Collaborate with the Spiritual Care Team: Inform nurses about the patient’s spiritual or religious beliefs and preferences. Be open to using provided spiritual resources (e.g., reading a familiar prayer) to comfort the patient [29].

### 5.3. Recommendations for Assessing Patient Progress

To move beyond subjective impression and objectively evaluate the impact of holistic interventions, clinicians should consider integrating the following assessment tools into practice:

- Physical Wellness: Use the Activity of Daily Living (ADL) scale and the Six-Minute Walk Test (6MWT) to quantitatively measure functional recovery and mobility [25].
- Psychological and Emotional Wellness: Utilize short, validated tools like the Hospital Anxiety and Depression Scale (HADS) [25] for mood or the Impact of Events Scale-Revised (IES-R) for post-traumatic stress symptoms to track psychological recovery.
- Spiritual Wellness: The Functional Assessment of Chronic Illness Therapy—Spiritual Well-Being (FACIT-Sp) scale is a brief, validated instrument suitable for assessing spiritual well-being in clinical settings [30].
- Overall Progress: Simple 0–10 numeric rating scales (NRS) for patient-reported pain, stress, and well-being can be administered quickly before and after interventions like CRC to gauge immediate effect [26].

### 5.4. Directions for Future Research

The studies open various avenues for further investigation, critical for advancing knowledge in this domain. One significant area for future research is exploring the long-term psychological impacts of interventions like patient diaries after discharge [39,40,41,42,43]. This could provide insights into how such tools can be leveraged for ongoing support beyond the critical care setting.

Additionally, understanding family involvement in diverse populations could enhance the applicability of current findings and ensure that interventions are culturally sensitive and effective across different demographics. Lastly, conducting qualitative research to delve into the lived experiences of patients and families regarding spiritual care could enrich the evidence base for integrating spirituality into critical care practices, helping to elucidate how these dimensions affect recovery metrics.

Future studies should aim for larger, more diverse samples and longer follow-up periods to assess the sustained impact of these interventions on patient outcomes. Furthermore, exploring the existential aspects of spiritual well-being and how these affect patient satisfaction could provide deeper insights into the role of spirituality in healthcare. Fostering a culture of holistic care in ICUs that prioritizes spiritual well-being alongside physical and emotional health is paramount. Continued research and the development of structured, evidence-based spiritual care practices are essential for improving patient outcomes and enhancing the quality of care in critical settings. By addressing these gaps, the healthcare community can better meet the diverse needs of patients, ultimately leading to improved health outcomes and quality of life. Future studies should focus on developing and testing standardized bundles of holistic care interventions. Research must prioritize robust methodologies: larger, multi-center randomized controlled trials (RCTs) with longer follow-up periods, explicit blinding procedures, and the use of consistent, validated outcome measures across the six wellness dimensions to allow for future meta-analysis.

#### Limitations of the Review

This systematic review is subject to several limitations. First, the inclusion of only seven studies restricts the breadth of evidence and may limit the generalizability of the findings. While this may reflect the specificity of the topic and the stringent inclusion criteria applied, it necessitates a more cautious interpretation of the conclusions. Importantly, the methodological quality of the included studies, as appraised by JBI tools (Table 4) and discussed below, introduces further constraints on the robustness of our findings.

A critical appraisal of the included studies reveals common limitations that impact the validity of the evidence. The frequent use of single-center designs raises concerns about external validity, as the findings may be influenced by local practices, cultures, and patient populations, limiting their generalizability to other ICU settings. Furthermore, several studies featured small sample sizes, which reduces statistical power and increases the risk of Type II errors, potentially obscuring true intervention effects.

The absence of control groups in observational and quasi-experimental studies significantly threatens internal validity, making it difficult to attribute observed outcomes solely to the intervention by ruling out confounding factors or natural recovery over time. Additionally, the heavy reliance on self-reported outcomes for measures of psychological and spiritual well-being introduces the potential for social desirability and recall bias. The lack of blinding in several trials further compounds these issues, as expectations of participants and caregivers could influence the results.

The heterogeneity in study designs, sample sizes, and outcome measures posed challenges for direct comparison and precluded meta-analytical synthesis. Furthermore, the methodological heterogeneity of the included studies necessitated a narrative synthesis, which, while providing rich qualitative insights, precludes the drawing of definitive quantitative conclusions regarding the magnitude of effects. This limits the ability to make robust comparative inferences about the efficacy of different interventions. Many of the studies were conducted in single-center settings with limited sample populations, which may introduce selection bias and affect external validity. Additionally, several interventions relied on self-reported outcomes, which are inherently prone to bias. Notably, while the review aimed to explore all six dimensions of wellness, the available evidence was heavily skewed. The scarcity of data on social and environmental wellness directly reflects a significant gap in the current research landscape. Our search strategy and inclusion criteria were designed to be broad and inclusive of these domains (e.g., using terms like “social” and “environmental”). However, the retrieved studies that met our eligibility criteria predominantly evaluated interventions targeting physical, psychological, emotional, and spiritual outcomes. This suggests that social and environmental interventions are underrepresented in the interventional literature for ICU patients, highlighting a critical area for future primary research. Finally, the search strategy was built around the specific concept of “holistic care” and its operationalization through the six dimensions of wellness. While related, the broader established concept of “patient-centered care” was not used as a primary search term. This focused approach ensured the review remained aligned with its theoretical framework but may have resulted in the omission of some relevant interventions primarily described under the patient-centered care paradigm.

## 6. Conclusions

The limited but promising evidence indicates that holistic care interventions may contribute positively to ICU patient recovery and highlights a critical area for future research. This systematic review synthesized emerging evidence on holistic care interventions for critically ill patients, framed around the six dimensions of wellness. The findings from the seven included studies suggest that such interventions hold promise for positively influencing patient recovery in the ICU. Specifically, the reviewed evidence provides preliminary support for the potential benefits of interventions targeting physical (e.g., corporeal rehabilitation, early intensive rehab), psychological and emotional (e.g., patient diaries, family involvement, reflexology), and spiritual (e.g., structured spiritual care, toolkits) wellness.

However, this review also underscores significant gaps in the current evidence base. Notably, no studies were identified that primarily investigated interventions designed for social wellness (e.g., building new support networks) or environmental wellness (e.g., structural modifications to the ICU environment), highlighting crucial areas for future research. The conclusions of this review are necessarily constrained by the limited number of studies (n = 7), their methodological heterogeneity, and the inherent limitations of the primary research, including small sample sizes and a reliance on single-center designs. Therefore, the synthesized evidence should be interpreted as exploratory and indicative of potential areas for innovation rather than as definitive proof of efficacy.

Notwithstanding these limitations, the collective findings emphasize the necessity of a multi-dimensional approach to critical care recovery. The insights garnered can inform both clinical practice and healthcare education, guiding the development of training programs and care protocols that address the complex, interrelated needs of ICU survivors. Future robust, multi-center research with longer follow-up periods is essential to validate these preliminary findings, determine generalizable effects, and establish standardized bundles of holistic care that seamlessly integrate all six dimensions of wellness into standard ICU practice.

## Figures and Tables

**Table 1:** SPICE Framework and Definitions.

Focus	Conceptual Question	Features
Setting (S)	Where is it?	Inclusion: Critical care settings (e.g., intensive care unit, critical care unit); Exclusion: Pediatric ICUs, coronary care units, general wards, end-of-life care.
Perspective (P)	Who is affected?	Inclusion: Critically ill adult patients (>18 years) admitted to critical care units. Exclusion: Pediatric patients, cardiovascular patients, palliative/terminally ill patients.
Intervention (I)	What is the intervention?	Inclusion: Interventions related to holistic care (physical, emotional, psychological, social, spiritual, environmental). Exclusion: Interventions targeted at healthcare professionals.
Comparison (C)	What is compared?	Standard nursing care.
Evaluation (E)	What outcomes?	Patient outcomes relating to the six dimensions of wellness within holistic care.

**Table 2:** Example of Database Searches.

Database	Boolean Search Strategy
PubMed	(“ICU” OR “intensive care” OR “critical care”) AND (“holistic nursing care” OR “holistic nursing practice” OR “holistic nursing care practice” OR holistic *) AND (patient outcome OR outcome * OR physical * OR emotional * OR psychological * OR spiritual * OR social * OR environmental *)
Scopus	(“holistic AND nursing AND care” OR “holistic AND nursing AND practice” OR “holistic AND nursing AND care AND practice” OR holistic *)
Web of Science	(“ICU” OR “intensive care” OR “critical care”) AND (“holistic nursing care” OR “holistic nursing practice” OR “holistic nursing care practice” OR holistic *) AND (patient outcome OR outcome * OR physical * OR emotional * OR psychological * OR spiritual * OR social * OR environmental *)

**Table 3:** Characteristics of Included Studies.

Author (Year) Country	Study Design	Population and Setting	Intervention	Primary Wellness Dimension(s)	Key Outcomes Measured	Main Findings
Bourgeon-Ghittori et al. (2022) France	Observational	297 critically ill patients (median age 59, 44% male) ICU setting	**Corporeal Rehabilitation Care (CRC):** Multi-sensory, esthetic care sessions delivered by socio-estheticians.	Physical, Emotional	Pain, stress, well-being (NRS) Dyspnea, respiratory rate 28-day mortality	Significant immediate improvements in stress, pain, and well-being (*p* < 0.001). Improvements in dyspnea and respiratory rate (*p* = 0.004). CRC responders had significantly lower 28-day mortality (4% vs. 15%, *p* = 0.005). High patient satisfaction (median score 10/10).
Black et al. (2011) UK	Quasi-experimental (Comparative time series)	170 patients and families General ICU	**Structured Family Involvement:** Nurses facilitated family participation using a guidance booklet for psychological care.	Psychological, Social	Incidence of delirium (ICDSC) Psychosocial recovery (SIP at 4, 8, 12 weeks)	No significant reduction in delirium incidence. Significantly better psychosocial recovery scores at all time points in the intervention group (large effect sizes, η^2^ = 0.37–0.40).
Kincheloe et al. (2018) USA	Quasi-experimental	54 nurses, 132 patients and family members Two acute care units	**Spiritual Care Toolkit:** Provided multi-faith resources (books, music, journals) to patients, families, and nurses.	Spiritual	Spiritual perspectives (SPS) Toolkit usefulness surveys	Significant disparity between nurse and patient/family spiritual perspectives. Toolkit effectively helped nurses overcome barriers to spiritual care delivery. 62–75% of patients/families found toolkit resources helpful.
Lemyze et al. (2022) France	Retrospective single-center	50 tracheostomized COVID-19 survivors Post-ICU weaning center	**Early Intensive Rehabilitation:** Combined physical rehab with a structured decannulation protocol.	Physical, Psychological	Frailty, ADL score 6 min walk test (6MWT) Anxiety/Depression (HADS)	Significant improvements in ADL (0 to 6), 6MWT distance (0 to 253 m), frailty (7 to 3), and HADS scores (18 to 10) (all *p* < 0.001). 96% of patients were decannulated and discharged home.
Pattison et al. (2019) UK	Mixed-methods	50 patients Critical care units	**Patient Diaries:** Diaries maintained by staff/family during ICU stay and given to patients post-discharge.	Psychological, Emotional	PTSS-14, EQ-5D-3L Diary evaluation questionnaire	95% found diaries helpful; 90% reported they helped fill memory gaps. Qualitative data highlighted value in reconstructing narrative and emotional processing. No significant quantitative changes in PTSS-14 or EQ-5D scores.
Korhan et al. (2014) Turkey	Randomized Controlled Trial (RCT)	60 mechanically ventilated patients ICU setting	**Reflexology:** 30 min sessions (foot, hand, ear) twice daily for 5 days.	Physical, Emotional	Vital signs (BP, HR, RR) Sedation needs (AACNSAS)	Significant reduction in physiological signs of anxiety (BP, HR, RR; *p* < 0.001). Significant improvements in agitation, anxiety, sleep, and patient-ventilator synchrony. Lower sedation requirements in the intervention group.
Bulut et al. (2023) Turkey	Randomized Controlled Trial (RCT)	64 patients (32/group) ICU setting	**Structured Spiritual Care:** 8 sessions based on the T.R.U.S.T. model over 4 weeks.	Spiritual	Spiritual Well-Being Hope, Loneliness Life Satisfaction	Significant increases in spiritual well-being, hope, and life satisfaction in the intervention group (*p* < 0.001). Significant decreases in emotional and social loneliness (*p* < 0.001). No significant changes in the control group.

Abbreviations: NRS: Numeric Rating Scale; ICDSC: Intensive Care Delirium Screening Checklist; SIP: Sickness Impact Profile; SPS: Spiritual Perspective Scale; ADL: Activity of Daily Living; 6MWT: 6-Minute Walk Test; HADS: Hospital Anxiety and Depression Scale; PTSS-14: Post-Traumatic Stress Symptoms-14; EQ-5D-3L: EuroQol 5-Dimensions 3-Level; AACNSAS: American Association of Critical-Care Nurses Sedation Assessment Scale; BP: Blood Pressure; HR: Heart Rate; RR: Respiratory Rate.

**Table 4:** Results of the methodological quality appraisal using Joanna Briggs Institute (JBI) tools.

Author (Year)	Study Design	JBI Checklist	Total Score	Summary of Appraisal & Key Limitations
Bourgeon-Ghittori et al. (2022) [26]	Observational	Checklist for Analytical Cross-Sectional Studies	6/8	Moderate quality. Strengths: Clearly defined criteria, standardized measurement. Key limitations: The lack of a control group significantly limits the ability to attribute outcomes solely to the intervention, as confounding factors and natural recovery cannot be ruled out.
Black et al. (2011) [24]	Quasi-Experimental	Checklist for Quasi-Experimental Studies	7/9	Moderate quality. Strengths: Clearly defined groups, complete follow-up. Key limitations: The non-randomized allocation of participants introduces a high risk of selection bias, weakening causal inferences. Blinding was not used.
Kincheloe et al. (2018) [27]	Quasi-Experimental	Checklist for Quasi-Experimental Studies	6/9	Moderate quality. Strengths: Multiple outcome measurements from different perspectives. Key limitations: The absence of a control group makes it difficult to assess the toolkit’s effect compared to standard care. No blinding or allocation concealment was implemented.
Lemyze et al. (2022) [25]	Case Series	Checklist for Case Series	6/10	Low to moderate quality. Strengths: Complete participant inclusion for the cohort, clear reporting of demographics. Key limitations: As a single-arm case series with no comparator, the design is highly susceptible to bias and confounding. Outcomes were not independently assessed.
Pattison et al. (2019) [28]	Mixed-Methods	Checklist for Mixed Methods Research	7/8 (Qual) 7/8 (Quan)	Good quality. Strengths: Methodological components are well-integrated to address the research question, and both qualitative and quantitative elements scored well. Key limitations: The study does not explicitly state how divergences between qualitative and quantitative findings were addressed.
Korhan et al. (2014) [29]	Randomized Controlled Trial (RCT)	Checklist for RCTs	8/13	Moderate quality. Strengths: Randomization was used, and groups were similar at baseline. Key limitations: The high risk of performance bias as participants and therapists were not blinded. Detection bias is also a concern as the outcome assessor was not blinded.
Bulut et al. (2023) [30]	Randomized Controlled Trial (RCT)	Checklist for RCTs	9/13	Good quality. Strengths: Proper randomization, complete follow-up, and reliable outcome assessment. Key limitations: The lack of blinding of participants and therapists (performance bias) is a notable limitation, as expectations could influence the results of interventions like spiritual care.
